# Creating an educationally minded schedule: one approach to minimize the impact of duty hour standards on intern continuity clinic experience

**DOI:** 10.1007/s40037-014-0117-0

**Published:** 2014-05-20

**Authors:** Dominick DeBlasio, M. Kathleen Kerrey, Heidi Sucharew, Melissa Klein

**Affiliations:** 1 Division of General and Community Paediatrics, Cincinnati Children’s Hospital Medical Center, 3333 Burnet Ave, MLC 2011, Cincinnati, OH 45229 USA; 2 Division of Biostatistics and Epidemiology, Cincinnati Children’s Hospital Medical Center, Cincinnati, OH USA

**Keywords:** Continuity clinic, Accreditation Council of Graduate Medical Education, Duty hour standards, Alternative day clinics

## Abstract

To determine if implementing an educationally minded schedule utilizing consecutive night shifts can moderate the impact of the 2011 duty hour standards on education and patient continuity of care in longitudinal primary care experience (continuity clinic). A 14-month pre–post study was performed in continuity clinic with one supervising physician group and two intern groups. Surveys to assess attitudes and education were distributed to the supervising physicians and interns before and after the changes in duty hour standards. Intern groups’ schedules were reviewed for the number of regular and alternative day clinic (i.e. primary care experience on a different weekday) sessions and patient continuity of care. Fifteen supervising physicians and 51 interns participated (25 in 2011, 26 in 2012). Intern groups’ comfort when discussing patient issues, educational needs and teamwork perception did not differ. Supervising physicians’ understanding of learning needs and provision of feedback did not differ between groups. Supervising physicians indicated a greater ability to provide feedback and understand learning needs during regular continuity clinic sessions compared with alternative day clinics (all *p* < 0.05). No significant difference was detected between intern groups in the number of regularly scheduled continuity clinics, alternative day clinics or patient continuity of care. The 2011 duty hour standards required significant alterations to intern schedules, but educationally minded scheduling limited impact on education and patient continuity in care.

## Introduction

Longitudinal ambulatory experience, or continuity clinic, is a critical element of paediatric residency training, providing opportunities to develop relationships with patients and families while observing the impact of interventions on growth, development and illness [1]. Development of long-term, resident-supervising physician relationships has been shown to correlate with increased resident satisfaction due to consistent provision of modelling of medical decision-making, interpersonal communication and feedback [2]. In the United States (US), this experience was mandated by the Accreditation Council of Graduate Medical Education (ACGME) and most residents spend half a day per week in continuity clinic during each year of training [1]. Other nations and specialities have utilized longer, continuous blocks of ambulatory care, as opposed to a weekly continuity experience throughout the entire residency training period, in order to maximize learning and satisfaction in the ambulatory setting and minimize the fragmentation of care [3, 4].

In 2003, the ACGME duty hour standards eliminated continuity clinic following a 24-h on-call shift, but continued to require a minimum of 36 continuity clinic shifts annually [5]. Difficulty in completing mandated shifts led to development of alternative day clinics, i.e. continuity clinic on a different weekday with potentially different supervising physicians and co-residents. Residents previously reported that the quality of teaching and learning, level of comfort and autonomy may differ on alternative day clinics [5]. Supervising physicians reported that working with alternative day residents complicated the supervising physicians’ ability to provide learner-centred teaching and hindered efforts at monitoring progress for these residents [5]. In 2011, ACGME regulations restricted interns to a maximum of 16 continuous hours [6] necessitating shift work and additional nights. This change potentially interfered with continuity clinic due to elimination of pre- and post-night shift continuity clinic. With the 36 session requirement unchanged [7], speculation of increased alternative day clinics emerged, raising concerns about impact on continuity clinic experience [8, 9]. Various specialities have developed different schedules to address the effects of duty hour standards on continuity clinic experience [3]. This study, utilizing surveys and chart review, was developed to determine if implementation of an educationally minded schedule, utilizing consecutive night shifts instead of night shifts equally spaced throughout the month, could minimize effects on education, overall satisfaction and patient continuity.

## Methods

This pre–post study was conducted from May 2011 through June 2012 with one supervising physician group and two intern groups before and after the 2011 changes in duty hour standards. It was approved as an exempt study by Cincinnati Children’s Hospital Medical Center (CCHMC) Institutional Review Board.

### Setting and subjects

The study was performed in the Paediatric Primary Care Centre (PPCC), a large, academic, urban outpatient primary care centre. PPCC is the continuity clinic site for approximately 75 residents annually. In their first year, interns are assigned a regular continuity clinic day of the week. If an intern is at risk of falling below the 36 required continuity clinic sessions, alternative day clinics are scheduled.

The July 2010–June 2011 interns (Group A) comprised the pre-duty hour standard change group. The July 2011–June 2012 interns (Group B) comprised the post-group. The supervising physicians were the same for both groups.

### Study design

Surveys were distributed to both intern groups in May of their intern year. Supervising physician surveys were distributed before the changes in duty hour standards in May 2011 and after the changes in May 2012.

Continuity clinic schedules were accessed from the electronic medical record. The number of continuity clinic sessions scheduled on regular and alternative days for the entire year was measured for both intern groups. A continuity patient was defined as a patient seen ≥2 times by the same intern for any issue. The number of continuity patients seen by each intern was determined by reviewing patient schedules from February through June in both years. Every patient chart from this 5-month period was reviewed; if the patient had been seen by the same intern earlier that academic year, she/he was considered a continuity patient.

### Survey development

Two surveys, one for interns and one for supervising physicians, were developed to identify demographic information and assess continuity clinic experience. The intern survey included Likert-based assessments of teamwork, educational experience, patient care issues and concerns about duty hour standards. The supervising physician survey included Likert-based questions to assess changes before and after implementation of the new duty hour restrictions in perception of feedback, education and patient care when working with interns during both regular continuity clinic and alternative day clinics. Surveys were developed by clinician educators from the Division of General and Community Paediatrics at CCHMC and reviewed by experts from the Division’s Education Section for content validity. Surveys were completed on an internet survey platform.

### Statistical analysis

Demographic differences between intern groups were assessed using Chi square statistics (gender) or Fisher’s exact test (age, ethnicity). Differences in Likert-based responses (1 = strongly disagree to 5 = strongly agree) for teamwork, educational experience and issues relating to patient care were assessed using the Wilcoxon rank-sum test. Differences in the proportion indicating no concerns with the new duty hour requirements were assessed using Fisher’s exact test.

The Wilcoxon signed-rank test was used to evaluate changes in supervising physicians’ perceptions of the educational experience and differences by individual supervising physicians comparing regular day and alternative day clinics. A shift in the proportion indicating no concerns regarding the changes in duty hour standards pre- and post-implementation was assessed using McNemar’s test.

Differences between groups in the number of continuity clinic sessions scheduled on regular and alternative days and number of continuity patients were evaluated using two sample t-tests.

## Results

### Demographics

Twenty-five Group A and 26 Group B interns participated. Intern groups did not differ in regard to gender, age, or race/ethnicity. The same 15 supervising physicians completed surveys both years. The majority of supervising physicians were Caucasian, female and had been supervisors for at least 2 years.

### Intern survey

All interns completed surveys. There was no statistically significant difference between groups with respect to perceived comfort discussing patients with the supervising physician or teamwork with co-residents. Eight Group A interns (32 %) reported concern about potential impact of the new duty hour requirements on the continuity clinic experience compared with 3 (12 %) Group B interns (*p* = 0.1) (Table 1).

**Table 1:** Summary of intern and supervising physician survey responses before and after the duty hour standards (DHS) change

Intern survey questions regarding regular CC day	Group A, pre-DHS changes 2011 N = 25	Group B, post-DHS changes 2012 N = 26	*p* value
Comfortable discussing patients with supervising physicians	5 (4, 5)	5 (4, 5)	0.28
Teamwork with co-residents	2 (2, 4)	2 (1, 3)	0.27
Supervising physicians understand my educational needs	5 (4, 5)	4 (4, 5)	0.06
Supervising physicians provide me with sufficient feedback	4 (4, 5)	4 (3, 4)	0.09

Supervising physician survey questions regarding regular CC day	Pre-DHS changes 2011 N = 14**	Post-DHS changes 2012 N = 14**	*p* value
Comfortable providing feedback	5 (4, 5)	4 (4, 5)	0.73
Understand the learning needs of interns	4 (3, 5)	4 (4, 4)	1.0
Interns are comfortable approaching me about patient care issues	4 (4, 5)	4 (4, 5)	0.31
Interns are comfortable approaching me about educational needs	4 (3, 5)	4 (3, 4)	0.69

Supervising physician survey questions on differences between regular and alternative day CC days	Pre-DHS changes 2011 N = 14**			Post-DHS changes 2012 N = 14**		
Survey questions	Regular day	ADC	*p* value	Regular day	ADC	*p* value
Comfortable providing feedback	5 (4, 5)	3 (3, 4)	<0.01	4 (4, 5)	4 (3, 4)	0.03
Understand the learning need of interns	4 (3, 5)	3 (2, 3)	0.02	4 (4, 4)	3 (2, 4)	0.02

Data presented as median (25th percentile, 75th percentile) with Likert responses coded as 1 = strongly disagree to 5 = strongly agree

Difference (Diff) = Regular continuity clinic (CC) day minus alternative CC day Likert response

** One supervising physician did not complete all survey questions

### Supervising physician survey

All supervising physicians completed pre- and post-surveys, although one did not respond to all questions. No statistically significant differences were observed in supervising physicians’ perceived abilities to provide feedback, understand learning needs, or discuss patient care or educational issues on regularly scheduled continuity clinic sessions after implementation of the new duty hour standards. During both years, the supervising physicians indicated greater abilities to understand learning needs and provide feedback during regular continuity clinic sessions compared with alternative day clinics (Table 1). In 2011, prior to duty hour changes, 14 (93 %) of supervising physicians reported concerns about potential impact of the new duty hour requirements on continuity clinic experience, compared with 8 (53 %) 1 year later (*p* = 0.01).

### Chart review

All interns met the 36 session requirement. The mean number of regular continuity clinic sessions (34 vs. 35, *p* = 0.14) and alternative day sessions (3 vs. 2, *p* = 0.11) did not statistically differ between groups. The mean number of continuity patients seen (21 vs. 19; *p* = 0.41) was not statistically different between groups.

## Discussion

Implementation of an educationally minded schedule minimized the impact of the 2011 duty hour standards on interns’ longitudinal primary care experience. Reformatting the intern schedule to work 4–5 consecutive night shifts instead of 6–7 night shifts equally spaced throughout the month resulted in missing only one continuity clinic session per inpatient month, similar to the prior post-call continuity clinic experience and created fewer alternative day clinics than expected. We believe that since our schedule did not increase the number of alternative day clinics, neither interns nor supervising physicians perceived differences in teamwork, feedback, education or comfort discussing patient care or learning needs after the implantation of the new duty hour standards.

Continuity clinic supervising physicians serve as paediatric primary care educators, as well as role models for interpersonal communication and clinical decision-making indicating the broad impact of the longitudinal care experience [2]. The consistency in the supervising physician-resident relationship following the 2011 duty hour requirements differs from the experience following the 2003 duty hour standards which eliminated post-call clinic, resulting in the addition of alternative day clinics to meet the 36-session requirement. Studies following these changes showed supervising physicians felt they were less effective teachers and interns noted differences in education [5]. In contrast, supervising physicians and interns in our clinic reported no significant changes in education following 2011 duty hour standards implementation, likely reflective of limited changes in alternative day clinics.

Continuity with patients is critical in the primary care experience as it affords residents the opportunity to learn from the patients and families over an extended period of time and observe the effect of their interventions [1]. The importance of outpatient continuity education is not exclusive to US training programmes. Internationally, residency programmes also recognize the educational importance of patient continuity in the longitudinal primary care experience as this continuity is a key part in learning the impact of medical decisions and interventions on the course of illness and learning the different aspects of chronic disease management [4]. In the US, preservation of patient continuity may be unique to continuity clinic since continuity scheduling is a priority and discrete clinic sessions fit well within a shift work framework. However, prior to the new duty hour standards, concern for outpatient continuity surfaced, similar to concerns of disruption of inpatient continuous care raised by residents and programme directors [8, 9]. After implementation of the new duty hour standards, surgical residents reported decreased inpatient continuous care [10], and surveys of programme directors found ownership of patients and continuous care was worse with the new duty hour requirements [11]. In contrast, our residents did not experience changes in patient continuity within continuity clinic.

This study has limitations including a generous definition of a continuity patient and a small sample of residents at a single site within one US residency programme over a short period, thereby limiting power to find a difference and overall generalizability. Also, we chose to investigate the narrow focus of effect of duty hour standards on the continuity clinic experience and did not explore potential effects of consecutive night shifts on alertness or safety.

## Conclusion

The 2011 duty hour standards required alterations to resident schedules, but implementation of an educationally minded schedule limited the impact on perceptions of education, teamwork, feedback, understanding learning needs, and discussing patient care or educational issues in continuity clinic. The mean number of continuity clinic patients and sessions scheduled on interns’ regular clinic day did not significantly differ and relationships between interns, families and supervising physicians remained strong. This schedule minimized the impact of duty hours on the continuity clinic experience in our programme; however, we recognize it may not be adaptable to programmes of different sizes and complexity. Regardless, educationally minded scheduling is a viable way to preserve a longitudinal experience.
